# Prevalence of cognitive frailty and its associated factors in a population of Iranian older adults

**DOI:** 10.1007/s40520-024-02790-y

**Published:** 2024-06-21

**Authors:** Mohammad Javad Ghanbarnia, Seyed Reza Hosseini, Alijan Ahmadi Ahangar, Reza Ghadimi, Ali Bijani

**Affiliations:** 1 Social Determinants of Health Research Center, Health Research Institute, Babol University of Medical Sciences, Babol, Iran; 2 Department of Ophthalmology, Babol University of Medical Sciences, Babol, Iran; 3 Mobility Impairment Research Center, Health Research Institute, Babol University of Medical Sciences, Babol, Iran

**Keywords:** Frailty, Cognitive dysfunction, Depression, Disability, Dementia, Instrumental activities of daily living

## Abstract

**Background:**

In recent years cognitive frailty has emerged as an important predictor of adverse health outcomes in older adults. Herein, we aimed to investigate the prevalence and associated factors of cognitive frailty in a population of community-dwelling older adults in Iran.

**Method:**

This cross-sectional study was conducted as part of the second cycle of the Amirkola Health and Aging Project (AHAP). Physical frailty and cognitive impairment were evaluated using the FRAIL questionnaire and the mini-mental state examination (MMSE) respectively. Cognitive frailty was defined as co-existence of frailty and cognitive impairment without presence of dementia. Depression and disability were assessed using the Persian version of geriatric depression scale (GDS) and instrumental activities of daily living (IADL) questionnaire.

**Results:**

Overall 1775 individuals (47.1% female) with mean age of 69.7 ± 7.3 years were included in the final analysis. The prevalence of cognitive frailty was 12.0%. The prevalence of cognitive frailty among males and females was 4.3% and 20.7%, respectively. After adjusting for all possible confounders through binary logistic regression analysis, factors such as older age (OR 1.06, CI 1.03–1.09), female gender (OR 2.25, CI 1.42–3.57), illiteracy (OR 3.84, CI 2.03–8.29), more comorbidities (OR 1.21, CI 1.12–1.31), depression (OR 2.01, CI 1.40–2.86), and greater IADL disability (OR 1.68, CI 1.44–3.96), were independently and significantly associated with cognitive frailty.

**Conclusion:**

In this population of Iranian older adults, prevalence of cognitive frailty was consistent with its estimated mean global prevalence. Age, gender, illiteracy, comorbidities, depression and IADL disability were associated with cognitive frailty. Further research is required to develop screening tools and prevention strategies.

## Introduction

Cognitive frailty (CF) has gained substantial interest among researchers in recent years as an emerging predictor of older adults’ health outcomes. The term “cognitive frailty” was conceptualised after decades of research into cognitive impairment and frailty substantiated the significant bidirectional association, and often co-existence, of frailty and cognitive impairment [1]. The bidirectional association between cognitive impairment and frailty has been reported in both cross-sectional and longitudinal studies. Several studies have concluded that individuals with cognitive impairment are at increased risk of incident frailty and vice versa [2–5]. Moreover, it has been demonstrated that the combination of cognitive impairment and frailty increases the predictability of adverse health outcomes such as disability and mortality because of their cumulative effect [6, 7]. To address such concerns, in 2013, an international consensus group introduced the concept of cognitive frailty which was defined as concurrent cognitive impairment and physical frailty without presence of dementia [8].

Since its first introduction in 2013, cognitive frailty has been the topic of various cross-sectional and longitudinal studies to determine its risk factors and adverse consequences. A multitude of studies have demonstrated the detrimental effect of cognitive frailty on adverse health outcomes. Individuals with cognitive frailty are at increased risk of disability, hospitalisation, low quality of life, dementia and mortality [9–15]. Increased risk of mortality is particularly noteworthy because of its direct and indirect pathways. Cognitive frailty increases the risk of all-cause mortality not only directly [12, 14, 15], but also indirectly through diminished functional domains and disability [10, 11], dementia [13, 14] and increased risk of falls and its consequent injuries [16]. Additionally, those with cognitive frailty are more likely to be dependent on long term care, which in turn, can potentially increase the burden of this condition [17]. As a result, various studies have investigated the risk factors of cognitive frailty to further our knowledge on the etiology of this condition. Cross-sectional studies have demonstrated an array of factors significantly associated with cognitive frailty including age, education level, depression, social support and social interactions, physical activity, nutrition, disability or even eye diseases such as cataract [18–22]. However, the literature hasn’t been totally consistent regarding these risk factors and further research is required to corroborate these findings.

The prevalence of cognitive frailty varies from 1.7 to 40.0% in different populations [20, 21]. The inconsistency of these reports, is in part due to the lack of a gold standard operational definition and different diagnostic tools. Moreover, studies from various populations have demonstrated different factors associated with cognitive frailty. Although numerous studies have investigated this topic in east and southeast Asia and Europe, it has not been well studied in Iran. Evidently, findings from a certain population cannot be necessarily extrapolated to a different population due to the inherent geographic and ethnic differences of various populations. Previously, we conducted a study investigating the association between eye diseases and cognitive frailty in Iranian older adults [22]. To the best of our knowledge, there hasn’t been any other study regarding cognitive frailty in Iran. Hence, in this study we aimed to investigate the prevalence of cognitive frailty and its associated factors in a population of Iranian community-dwelling older adults.

## Methods

The data used in this study were collected during the second cycle of the Amirkola Health and Aging Project (AHAP) between 2016 and 2017. The AHAP is a comprehensive cohort study of older adults in northern Iran which investigates multidisciplinary geriatric conditions and its study protocols have been published elsewhere [23, 24]. All community-dwelling adults aged 60 years and older in this region were invited to partake in the study. All participants were informed of the details of the study and provided written informed consent. The study adhered to the tenets of the declaration of Helsinki and was approved by the ethics committee of Babol university of medical sciences (IR.MUBABOL.REC.1399.413).

All participants in the second cycle of the AHAP with available data on the variables of interest were included in this study. Overall 2135 individuals participated in the study out of which 1972 individuals had variables of interest available in our records. We further excluded individuals with prior confirmed history of stroke, neurodegenerative diseases such as Parkinson’s disease, dementia and those strongly suspicious of having dementia (i.e. MMSE < 18; moderate or severe cognitive impairment). In total, 1775 individuals were included for the final analysis.

Sociodemographic and medical history were collected through a series of standardized questionnaires and interviews administered by trained health care professionals. Anthropometric measurements and blood pressure were obtained through physical examinations. Body mass index (BMI, kg/m^2^) was derived from the equation of weight (Kg) divided by height (m) squared. BMI was further categorized into four groups; underweight (BMI < 18.5, kg/m^2^), normal (18.5≤BMI < 25.0, kg/m^2^), overweight (25.0≤BMI < 30.0, kg/m^2^) and obese (BMI≥30.0, kg/m^2^). Hypertension was defined as having prior confirmed diagnosis of hypertension, taking antihypertensive medications or high mean systolic (≥130 mmHg) or diastolic (≥80 mmHg) blood pressure in two consecutive visits. Fasting blood samples were drawn for laboratory measurements of total plasma concentrations of glucose, cholesterol (Chol, mg/dL) and Triglycerides (TG, mg/dL). Diagnosis of diabetes mellitus was based on prior confirmed diagnosis by a practitioner, taking anti-diabetic medications or two fasting plasma glucose concentrations > 125 mg/dL.

Chronic pain was assessed by asking the participants whether or not they had experienced pain for at least 3 months in the last 6 months. Participants were asked to rate their income level by answering the question “How do you rate your current earnings?” with five possible answers such as “very low, low, moderate, high and very high”. They were also asked to rate their self perceived health by answering the question, “compared to your peers, how do you rate your health status?” with five possible answers “bad, fair, good, very god, excellent”.

Depression was evaluated using the Persian version of the Geriatric Depression Scale (GDS-15) questionnaire. The reliability and validity of this questionnaire in assessing depression among Iranian older adults have been previously determined [25]. GDS-15 consists of 15 yes/no questions with a score ranging from 0 to 15 and higher scores indicating higher probability of depression. We further categorized depression severity into four subgroups based on GDS-15 scores; normal (0–4), mild (5–8), moderate (9–11) and severe (12–15). Subsequently, for the purposes of logistic regression analysis, individuals with mild or worse depression severity were classified as having depression.

Disability was assessed using the Persian version of Lawton’s Instrumental Activities of Daily Living (IADL) questionnaire [26, 27]. The original questionnaire evaluates 8 items such as communication (telephone), transportation, shopping, food preparation, housekeeping, doing laundry, taking medications and managing finances. Due to cultural considerations, three items such as food preparation, housekeeping and doing laundry were omitted from the questionnaire and five items consisting of communication (telephone), transportation, shopping, taking medications and managing finances were assessed. The level of performance for each task was scored on a 3-point scale as follows, “1. Unable to do”, “2. Needs some help” and “3. Completely independent”. The score for each item was then dichotomized as either 0 (complete independence in doing the task) or 1 (unable to do or needing some help). The total score ranged from 0 to 5 with higher scores corresponding to disability in more functional domains.

Cognitive function was tested using the Mini-Mental State Examination (MMSE). The Persian-translated and culturally adapted version of MMSE is a reliable tool for screening and estimating the severity of cognitive impairment which evaluates five cognitive domains such as orientation, registration, attention and calculation, recall and language [28, 29]. A score out of 30 was assigned for every participant with higher scores indicating better cognitive function. Participants with scores ≤ 23 were categorized as having cognitive impairment [29]. Individuals with prior confirmed diagnosis of dementia and those strongly suspicious of having dementia (i.e. MMSE < 18; moderate or severe cognitive impairment) were excluded from the study [30, 31].

Physical Frailty was determined using the Persian-translated version of the FRAIL questionnaire (FRAIL scale) which evaluates five frailty domains such as fatigue, resistance, ambulation, illness and loss of weight [32]. This questionnaire comprises five questions and total scores range from 0 to 5 with higher scores representing higher levels of frailty. Individuals with a score of zero were considered robust and scores of one or 2 represented prefrailty and those who scored ≥ 3 were considered frail.

Cognitive frailty (CF) was operationally defined based on the original definition introduced by the International Consensus Group on Cognitive Frailty in 2013 [8]. Individuals with concurrent physical frailty and cognitive impairment without a diagnosis of dementia were considered to have cognitive frailty. Accordingly, individuals with MMSE scores ≤ 23 and frailty scores ≥ 3 were determined to have CF. We further defined five more categories apart from cognitive frailty. These categories included robust (without CI, physical prefrailty or frailty), physical prefrailty only (without CI), physical frailty only (without CI), cognitive impairment only (without physical frailty or prefrailty), and CI + prefrailty (concurrent CI and physical prefrailty).

### Statistical analysis

Statistical analysis was performed using SPSS, version 26 (Statistical Package for Social Science, IBM- SPSS Inc, Chicago, USA). Continuous variables are presented using mean ± SD (standard deviation) and categorical variables are presented with n (number) and percentage. For the purposes of crude comparison of sociodemographic and clinical characteristics of participants, they were categorized into six groups based on their cognitive function and physical frailty status (as elaborated previously). To determine the factors associated with cognitive frailty, binary logistic regression was performed with cognitive frailty set as the dependent. The binary dependent variable consisted of either those with cognitive frailty or the rest of the study population without cognitive frailty. For the purposes of obtaining a more accurate result from the logistic regression analysis, all categorical independent variables were dichotomized. First, univariate and then multivariate logistic regression analysis were performed. Since age, gender and literacy were strongly associated with cognitive frailty, all univariate analysis were adjusted for these three variables. Finally, to determine all the factors independently associated with cognitive frailty after fully adjusting for all possible confounders, a stepwise backward LR (likelihood ratio) logistic regression was performed. Odds ratios are presented with their corresponding 95% CI (confidence interval). All p-values < 0.05 were considered statistically significant.

## Results

**Fig. 1:**
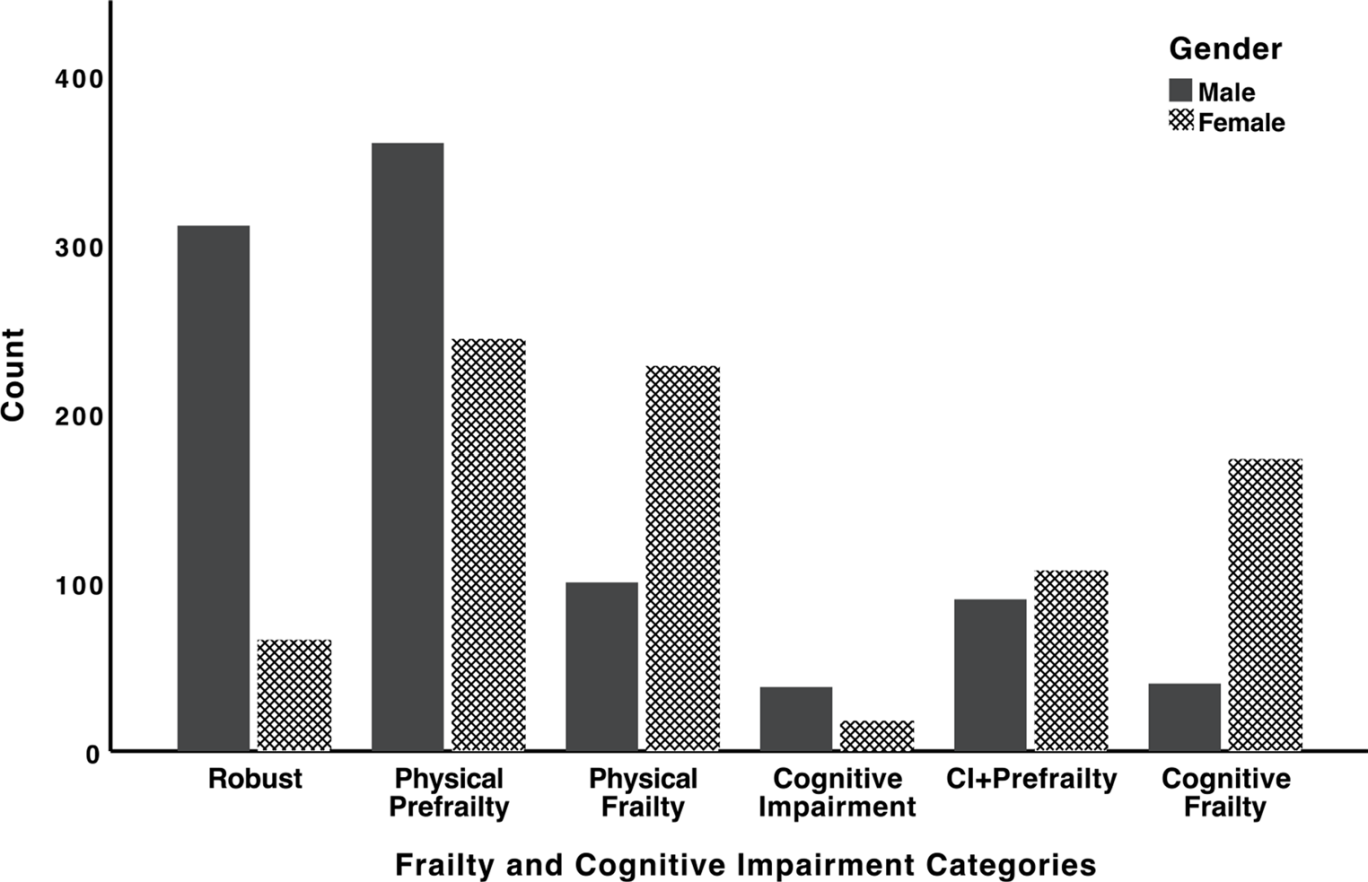
Comparison of the number of males vs. females across the six categories of frailty and cognitive impairment

In total 1775 individuals (mean age 69.72 ± 7.26 years) were included in the final analysis out of which 836 (47.1%) were female. Overall, the prevalence of cognitive frailty was 12.0% and prevalence of CF among male and female individuals was 4.3% and 20.7%, respectively. Cognitive frailty was remarkably more prevalent among illiterate individuals compared to literate individuals (19.8% vs. 1.6% respectively). The breakdown of sociodemographic and clinical characteristics of participants compared across the six frailty and cognitive impairment categories are presented in Table 1. A noticeable majority of those with CF were female (81.2%), while majority of robust individuals (82.5%) consisted of males (Table 1). On the other hand, majority (67.9%) of those with cognitive impairment alone were male (Table 1). Figure 1 further illustrates the comparison of the number of males vs. females across the six categories of frailty and cognitive impairment.

**Table 1:** Characteristics of the participants stratified based on various levels of physical frailty and cognitive impairment

	Overall (*n* = 1775)	Robust (*n* = 377)	Physical Prefrailty (*n* = 604)	Physical Frailty (*n* = 328)	Cognitive Impariment (*n* = 56)	CI + Prefrailty (*n* = 197)	Cognitive Frailty (*n* = 213)
Age, mean (y) ± SD	69.7 ± 7.3	67.4 ± 5.8	68.6 ± 6.7	70.1 ± 7.5	71.8 ± 7.6	71.6 ± 8.2	74.0 ± 7.2
*Age Category, n(%)*							
60–64	468 (26.4)	128 (34.0)	190 (31.5)	78 (23.8)	10 (17.9)	43 (21.8)	19 (8.9)
65–69	547 (30.8)	130 (34.5)	189 (31.3)	111 (33.8)	18 (32.1)	53 (26.9)	46 (21.6)
70–74	335 (18.9)	68 (18.0)	112 (18.5)	56 (17.1)	11 (19.6)	32 (16.2)	56 (26.3)
75–79	225 (12.7)	36 (9.5)	71 (11.8)	44 (13.4)	4 (7.1)	25 (12.7)	45 (21.1)
80–84	134 (7.5)	14 (3.7)	32 (5.3)	19 (5.8)	10 (17.9)	30 (15.2)	29 (13.6)
85–99	66 (3.7)	1 (0.3)	10 (1.7)	20 (6.1)	3 (5.4)	14 (7.1)	18 (8.5)
*Gender, n (%)*							
Male	939 (52.9)	311 (82.5)	360 (59.6)	100 (30.5)	38 (67.9)	90 (45.7)	40 (18.3)
Female	836 (47.1)	66 (17.5)	244 (40.4)	228 (69.5)	18 (32.1)	107 (54.3)	173 (81.2)
*Education level, n (%)*							
Illiterate	1013 (57.1)	113 (30.0)	282 (46.8)	199 (60.7)	50 (89.3)	168 (85.3)	201 (94.4)
Elementary	404 (22.8)	111 (29.4)	172 (28.5)	79 (24.1)	5 (8.9)	25 (12.7)	12 (5.6)
High school	241 (13.6)	92 (24.4)	105 (17.4)	40 (12.2)	0 (0.0)	4 (2.0)	0 (0.0)
Higher Education	116 (6.5)	61 (16.2)	44 (7.3)	10 (3.0)	1 (1.8)	0 (0.0)	0 (0.0)
*Literacy, n (%)*							
Literate	761 (42.9)	264 (70.0)	321 (53.2)	129 (39.3)	50 (89.3)	168 (85.3)	12 (5.6)
Illiterate	1013 (57.1)	113 (30.0)	282 (46.8)	199 (60.7)	6 (10.7)	29 (14.7)	201 (94.4)
*Living Alone*							
No	1612 (90.8)	360 (95.5)	559 (92.5)	294 (89.6)	50 (89.3)	170 (86.3)	179 (84.0)
Yes	163 (9.2)	17 (4.5)	45 (7.5)	34 (10.4)	6 (10.7)	27 (13.7)	34 (16.0)
*Self-rated income level*							
Very high	9 (0.05)	1 (0.3)	5 (0.8)	2 (0.6)	1(1.8)	0 (0.0)	0 (0.0)
High	39 (2.2)	14 (3.7)	11 (1.8)	7 (2.1)	2 (3.6)	2 (1.0)	3 (1.4)
Moderate	614 (34.6)	164 (43.7)	231 (28.2)	93 (28.4)	14 (25.0)	61 (31.0)	51 (23.9)
Low	723 (40.8)	157 (41.9)	245 (40.6)	131(39.9)	24 (42.9)	86 (43.7)	80 (37.6)
Very low	388 (21.9)	39 (10.4)	112 (18.5)	95 (29.0)	15 (26.8)	48 (24.4)	79 (37.1)
*Self-rated health status*							
Bad	89 (5.0)	2 (0.5)	15 (2.5)	43 (13.1)	0 (0.0)	8 (4.1)	21 (9.9)
Fair	192 (10.8)	19 (5.0)	46 (7.6)	64 (19.5)	1 (1.8)	21 (10.7)	41 (19.2)
Good	659 (37.1)	108 (28.6)	227 (37.6)	156 (47.6)	11 (19.6)	62 (31.5)	95 (44.6)
Very good	530 (29.9)	144 (38.2)	198 (32.8)	39 (11.9)	27 (48.2)	77 (39.1)	45 (21.1)
Excellent	305 (17.2)	104 (27.6)	118 (19.5)	26 (7.9)	17 (30.4)	29 (14.7)	11 (5.2)
BMI, mean ± SD	28.2 ± 4.9	27.2 ± 3.8	27.9 ± 4.5	29.6 ± 5.5	26.4 ± 4.9	27.8 ± 5.1	29.7 ± 5.6
*Body Mass Index*							
Underweight	22 (1.2)	2 (0.5)	9 (1.5)	3 (0.9)	0 (0.0)	5 (2.5)	3 (1.4)
Normal	444 (25.0)	118 (31.3)	151(25.0)	61 (18.7)	23 (41.1)	53 (26.9)	38 (17.8)
Overweight	719 (40.5)	176 (46.7)	253 (41.9)	119 (36.4)	17 (30.4)	82 (41.6)	72 (33.8)
Obese	589 (33.2)	81(21.5)	191 (31.6)	144 (44.0)	16 (28.6)	57 (28.9)	100 (46.9)
GDS Score, mean ± SD	4.0 ± 3.5	2.2 ± 2.5	3.4 ± 3.1	5.4 ± 3.6	2.8 ± 2.7	4.6 ± 3.5	6.4 ± 3.9
*Depression Severity*							
Normal (0–4)	1135(64.1)	317(84.3)	433(71.8)	159(48.5)	43(76.8)	107(54.3)	76(35.8)
Mild (5–8)	405(22.9)	46(12.2)	118(19.6)	97(29.6)	10(17.9)	61(31.0)	73(34.4)
Moderate (9–11)	159(9.0)	11(2.9)	38(6.3)	51(15.5)	2(3.6)	19(9.6)	38(17.9)
Severe (12–15)	73(4.1)	2(0.5)	14(2.3)	21(6.4)	1(1.8)	10(5.1)	25(11.8)
Medications, mean ± SD	3.7 ± 3.1	2.5 ± 2.6	3.4 ± 2.8	5.3 ± 3.4	1.6 ± 1.9	3.1 ± 2.8	5.2 ± 3.3
*Medications, n (%)*							
0	372(21.0)	120(31.8)	118(19.5)	41(12.5)	20(35.7)	51(25.9)	22(10.3)
1	185(10.4)	53(14.1)	75(12.4)	17(5.2)	12(21.4)	19(9.6)	9(4.2)
≥2	1218(68.6)	204(54.1)	411(68.0)	270(82.3)	24(42.9)	127(64.5)	182(85.4)
Comorbidities, mean ± SD	3.7 ± 2.2	2.2 ± 1.4	3.2 ± 1.8	5.2 ± 2.4	2.2 ± 1.5	3.6 ± 1.9	5.5 ± 2.2
*Comorbidities, n (%)*							
0	85 (4.8)	40 (10.6)	26 (4.3)	4 (1.2)	8 (14.3)	7 (3.6)	0 (0.0)
1	223 (12.6)	97 (25.7)	81(13.4)	9 (2.7)	13 (23.2)	18 (9.1)	5 (2.3)
2	305 (17.2)	94 (24.9)	109 (18.0)	38 (11.6)	15 (26.8)	33 (16.8)	16 (7.5)
≥ 3	1162 (65.5)	146 (38.7)	388 (64.2)	277 (84.5)	20 (35.7)	139 (70.6)	192 (90.1)
*Chronic Pain, n (%)*							
No	578 (32.6)	219 (58.1)	203 (33.6)	36 (11.0)	31 (55.4)	65 (33.0)	24 (11.3)
Yes	1197 (67.4)	158 (41.9)	401 (66.4)	292 (89.0)	25 (44.6)	132 (67.0)	189 (88.7)
*Hypertension, n (%)*							
No	494 (27.9)	135 (35.8)	184 (30.5)	58 (17.7)	25 (45.5)	61 (31.1)	31 (14.6)
Yes	1278 (72.1)	242 (64.2)	420 (69.5)	270 (82.3)	30 (54.5)	1278 (72.1)	181 (85.4)
*Diabetes*							
No	1192 (67.2)	284 (75.3)	422 (69.9)	181 (55.2)	49 (87.5)	134 (68.0)	122 (57.5)
Yes	582 (32.8)	93 (24.7)	182 (30.1)	147 (44.8)	7 (12.5)	63 (32.0)	90 (42.5)
*Fall (in the past year)*							
No	1400(78.9)	327(86.7)	502(83.1)	242(73.8)	45(80.4)	147(74.6)	137(64.3)
Yes	375(21.1)	50(13.3)	102(16.9)	86(26.2)	11(19.6)	50(25.4)	76(35.7)
IADL Score, mean ± SD	1.9 ± 1.5	1.0 ± 1.0	1.5 ± 1.2	2.3 ± 1.5	2.4 ± 1.1	2.6 ± 1.3	3.6 ± 1.3
TG mg/dL, mean ± SD	147.2 ± 85.2	138.6 ± 75.4	142.3 ± 74.8	155.9 ± 93.9	132.0 ± 116.0	152.0 ± 97.2	162.4 ± 91.2
Chol mg/dL, mean ± SD	191.4 ± 47.6	190.0 ± 46.4	188.3 ± 44.9	189.4 ± 50.5	194.4 ± 55.9	199.5 ± 46.4	197.7 ± 50.5

CI, cognitive impairment; BMI, body mass index; GDS, geriatric depression scale; IADL, instrumental activities of daily living; TG, triglycerides; Chol, cholesterol

**Fig. 2:**
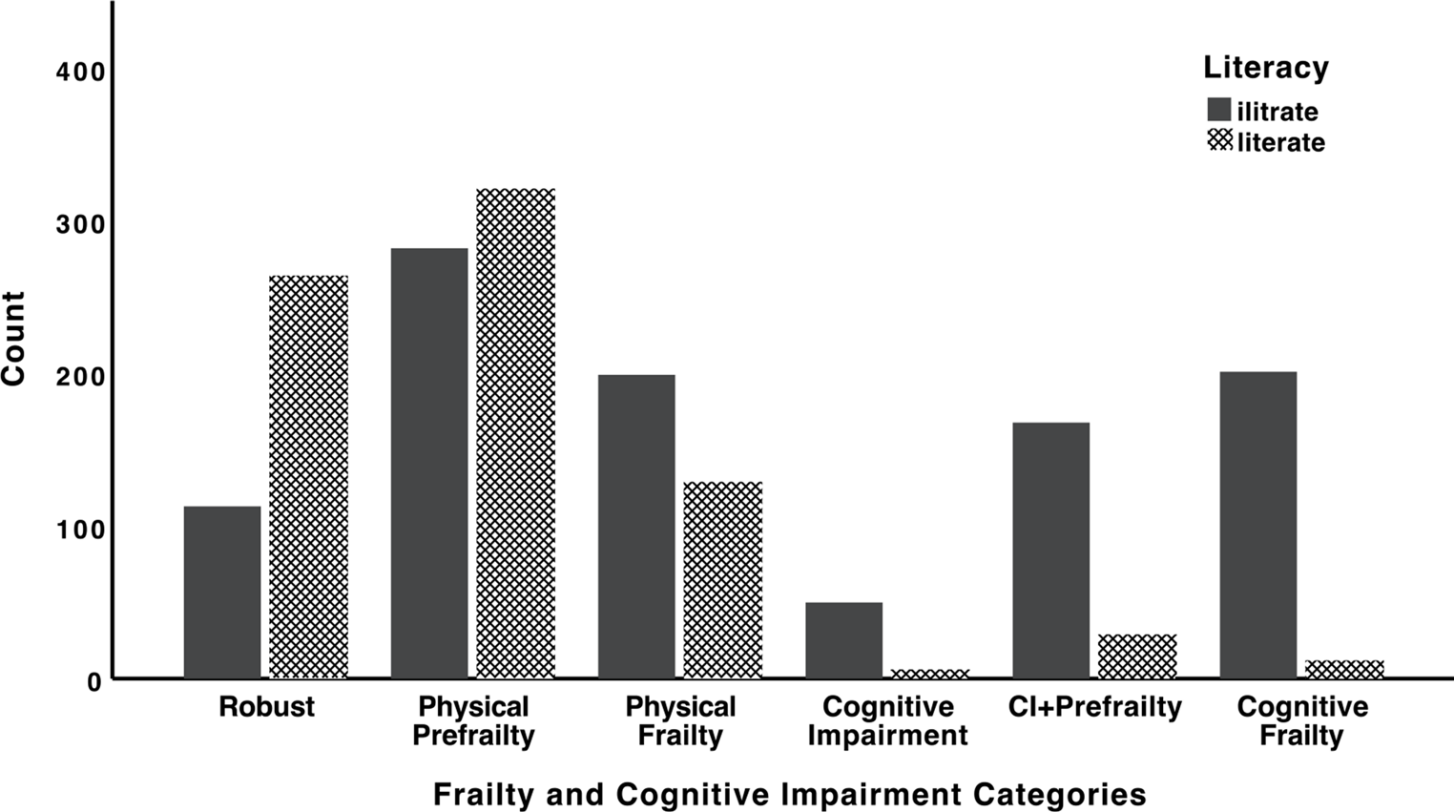
Comparison of the number of literate vs. illiterate individuals across the six categories of frailty and cognitive impairment

**Fig. 3:**
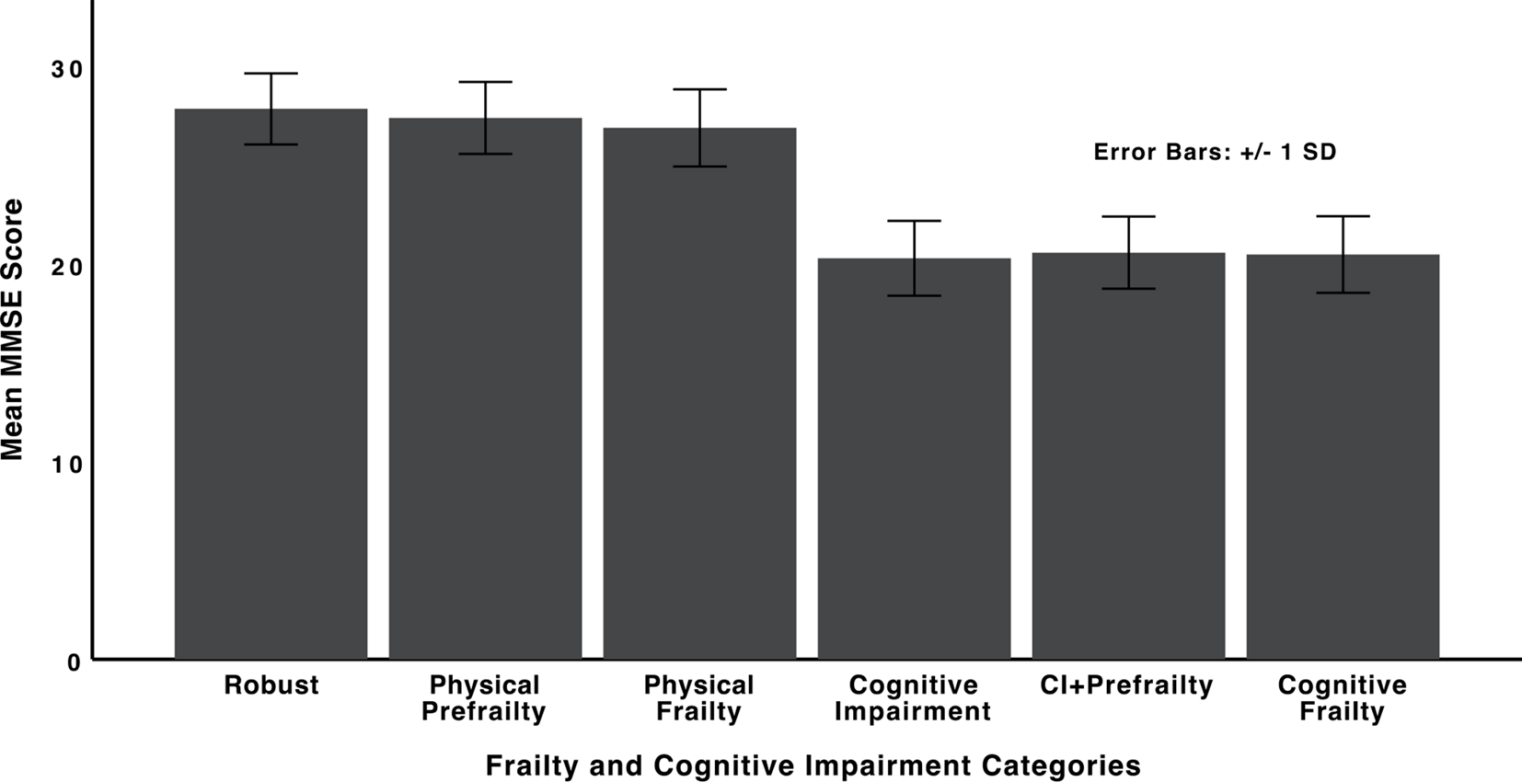
Comparison of mean MMSE scores across the six categories of frailty and cognitive impairment

Figure 2 compares the number of illiterate and literate individuals across the six categories of frailty and cognitive impairment. While 30.0% of robust individuals were illiterate, 94.4% of those with cognitive frailty were illiterate (Fig. 2). Figure 3 presents the comparison of mean MMSE scores across the six categories of frailty and cognitive impairment. Highest mean MMSE score belonged to the robust category (27.83 ± 1.80), while those with cognitive impairment only, had the lowest mean MMSE score (20.29 ± 1.90). Mean MMSE score for the cognitive frailty category was 20.48 ± 1.94.

Binary logistic regression was performed to determine the association of sociodemographic and clinical characteristics with cognitive frailty. The odds ratios of every characteristic was first computed by univariate logistic regression analysis adjusted for three main confounders; age, gender and literacy (Table 2). The variables of age, gender and literacy are three of the most notable confounders as determined by various studies. Furthermore, we also observed a strong association of age (OR 1.09, CI 1.06–1.11), gender (female; OR 6.84, CI 4.60-10.17) and illiteracy (OR 8.50, CI 4.53–15.61) with cognitive frailty in a logistic regression model adjusted for these three variables. Hence, we adjusted the univariate regression analysis of each characteristic with these three confounders before proceeding to the multivariate analysis. Lastly, in a multivariate logistic regression model fully adjusted for all confounders, age (OR 1.06, CI 1.03–1.09), female gender (OR 2.25, CI 1.42–3.57), illiteracy (OR 3.84, CI 2.03–8.29), depression (OR 2.01, CI 1.40–2.86), more comorbidities (OR 1.21, CI 1.12–1.31) and greater IADL disability (OR 1.68, CI 1.44–1.96), were independently and significantly associated with cognitive frailty (Table 3). However, living alone, self-rated income level, self-rated health status, BMI, number of comorbidities, number of medications, chronic pain, hypertension, diabetes mellitus, having fallen in the past year, total cholesterol and triglycerides were not significantly associated with cognitive frailty.

**Table 2:** Odds ratio of sociodemographic and clinical characteristics obtained by Univariate binary logistic regression, adjusted for age, gender and literacy

Variables	OR	95%CI	*P*-value
Age	1.09	1.06–1.11	< 0.001
*Gender*			
Male	Reference		
Female	6.84	4.60-10.17	< 0.001
*Literacy*			
Literate	Reference		
Illiterate	8.50	4.63–15.61	< 0.001
*Living Alone?*			
No	Reference		
Yes	0.77	0.48–1.23	0.275
*Self-rated income level*			
Moderate to very high	Reference		
Low to very low	1.23	0.86–1.76	0.260
*Self-rated health status*			
Good, very good & excellent	Reference		
Bad to fair	1.79	1.23–2.61	0.002
*BMI*			
Under 25	Reference		
25 and over	1.52	1.00-2.30	0.052
*Depression (GDS)*			
No	Reference		
Yes	2.58	1.85–3.60	< 0.001
Number of Comorbidities	1.30	1.20–1.40	< 0.001
Number of Medications	1.10	1.04–1.15	< 0.001
*Chronic Pain*			
No	Reference		
Yes	2.17	1.35–3.47	0.001
*Hypertension*			
No	Reference		
Yes	1.81	1.18–2.80	0.007
*Diabetes Mellitus*			
No	Reference		
Yes	1.45	1.04–2.02	0.030
*Falls (past year)*			
No	Reference		
Yes	1.62	1.14–2.29	0.007
Disability (IADL Score)	1.82	1.57–2.11	< 0.001
*TG mg/dL*			
<150	Reference		
≥150	1.30	0.93–1.82	0.120
*Chol mg/dL*			
<200	Reference		
≥200	1.11	0.80–1.53	0.544

OR, odds ratio; CI, confidence interval; BMI, body mass index; GDS, geriatric depression scale; IADL, instrumental activities of daily living; TG, triglycerides; Chol, cholesterol

**Table 3:** Odds ratios of factors significantly associated with cognitive frailty after fully adjusting for all possible confounders obtained by stepwise backward LR (likelihood ratio) logistic regression analysis

Variables	OR	95%CI	*P*-value
Age	1.06	1.03–1.09	< 0.001
*Gender*			
Male	Reference		
Female	2.25	1.42–3.57	< 0.001
*Literacy*			
Literate	Reference		
Illiterate	3.84	2.03–8.29	< 0.001
*Depression (GDS)*			
No	Reference		
Yes	2.01	1.40–2.86	< 0.001
Number of Comorbidities	1.21	1.12–1.31	< 0.001
Disability (IADL Score)	1.68	1.44–1.96	< 0.001

OR, odds ratio; CI, confidence interval; GDS, geriatric depression scale; IADL, instrumental activities of daily living

## Discussion

In this study we investigated the prevalence of cognitive frailty and its associated factors in a population of Iranian older adults. We found that the prevalence of cognitive frailty was 12.0%. Owning to the recent introduction of this concept and the lack of other pertinent research on this topic in Iran, substantiating our findings is challenging and requires further research. A multitude of studies have investigated this topic around the world and the reported prevalence of cognitive frailty varies substantially from 1.6% in South Korea [20] to 40.0% in Thailand [21]. The reported prevalence in this study is consistent with the findings from studies conducted in Singapore, South Korea, Peru and Taiwan which reported 10.7%, 11.2%, 11.3% and 15.8%, respectively [11, 15, 31, 33]. Furthermore, meta-analysis studies have estimated the global pooled prevalence of cognitive frailty to be between 9.0% and 16% [34, 35]. This indicates that the prevalence of cognitive frailty in this population of Iranian older adults (12%) is consistent with the global estimates. Inherent differences between various populations such as differences in geographical, ethnic, cultural and sociodemographic characteristics are among the reasons for such variations in the reported prevalence. However, as consistently noted in several review studies [12, 13, 34–36], the absence of a universally accepted operational definition for cognitive frailty, along with variations in the diagnostic tools utilized, appears to be a more prominent contributing factor to the inconsistency of reported results. This highlights the importance of further research for constructing a gold-standard diagnostic tool.

In this study we demonstrated some of the sociodemographic and clinical factors that are associated with cognitive frailty. In the fully adjusted logistic regression model, individuals with older age, female gender, illiteracy, higher number of comorbidities, depression and disability in more IADL functional domains were more likely to have cognitive frailty. We indicated that every one year increase in age was associated with 6.0% increased odds of having cognitive frailty (OR 1.06, CI 1.03–1.09). Moreover, 48.9% of individuals 80 years and older had cognitive frailty which was the highest rate among all age groups. This is consistent with several studies which presented age as an independent risk factor of cognitive frailty. For instance, Kim et al. [37] reported that for every one year increase in age, the odds of having cognitive frailty would increase by 15% (OR 1.15). Griffiths et al. [21] demonstrated that individuals older than 80 years of age were four times more likely to have cognitive frailty than those 65–69 years old (OR 3.95). The impact of age has also been revealed in longitudinal studies as Tang et al. [38] concluded that older individuals are at increased odds (OR 1.15) of incident cognitive frailty in a time span of six years. On the other hand, literature is less consistent regarding the effect of gender on cognitive frailty. In this study we reported that female gender was significantly associated with cognitive frailty (OR 2.25, CI 1.42–3.57). Most studies haven’t found an association between gender and cognitive frailty. However, studies that reported such association concluded that female gender is independently associated with increased odds of cognitive frailty which is consistent with our results [33, 39, 40]. Moreover, previous studies which investigated frailty and cognitive impairment in Iran reported that both of these conditions were more prevalent among female Iranian older adults compared to males [41, 42], which further justifies the association of female gender with cognitive frailty in this study.

Level of education is a prominent risk factor for cognitive frailty. In this study, we indicated that illiterate individuals were more likely to have cognitive frailty (OR 3.84, CI 2.03–8.29) compared with literate individuals. The association between education and cognitive frailty has been consistently demonstrated in cross sectional studies [18, 39, 40, 43]. Moreover, in a longitudinal study, Lee et al. concluded that higher level of education is protective against incident cognitive frailty [44]. Level of education has been independently linked to both cognitive impairment and frailty, hence, its association with cognitive frailty is expected [45].

Higher number of comorbidities was another factor significantly associated with cognitive frailty in this study. For every additional comorbidity, the odds of having cognitive frailty increased by 21% (OR 1.21, CI 1.12–1.31). The literature hasn’t been conclusive regarding the role of comorbidities as a risk factor for CF. Several studies have reported the significant association between comorbidities and cognitive frailty [19, 37, 39, 46, 47]. For instance, Kim et al. [37] concluded that four or more comorbidities would increase the odds of cognitive frailty by five-fold (OR 5.12). In this study, the FRAIL scale was utilized to determine frailty, and comorbidities is among the five questions included in this criteria. Using this diagnostic tool could have contributed to the observation that people with comorbidities were more likely to have cognitive frailty. Nevertheless, studies that utilized different frailty diagnostic tool such as the Fried frailty phenotype also reported similar results [19, 37, 39, 46, 47], which further corroborates the association between comorbidities and cognitive frailty.

The relationship between depression and cognitive frailty is noteworthy. In this study we indicated that older adults with depression were twice more likely (OR 2.01, CI 1.40–2.86) to have cognitive frailty compared to those without depression. A multitude of studies have demonstrated the significant association between depression and cognitive frailty [1, 19, 21, 39, 48]. This association has also been corroborated in meta-analysis studies, as Yuan et al. reported that older adults with depression were three and a half times more likely (OR 3.6) to have cognitive frailty [35, 49]. Moreover, cognitive frailty can potentiate the adverse outcomes related to depression. For instance, Yi et al. [17] indicated that cognitive frailty in patients with depression tripled the odds of long term care (OR 3.16). Similarly, several longitudinal studies have investigated the impact of depression on cognitive frailty. In a study in China, Yuan et al. [46] reported that not only did depression increase the risk of developing cognitive frailty in individuals with physical frailty by 75% but it also reduced the chances of reversing cognitive frailty.

The relationship between depression and cognitive frailty is expected due to the independent effect of depression on both cognitive impairment and physical frailty. Depression has been determined to be bidirectionally associated with frailty in several meta-analysis studies [50, 51]. Furthermore, Lee et al. [52] concluded that individuals with concurrent frailty and depression were more likely (OR 3.5) to have cognitive impairment. This highlights the notable relationship between the three conditions of depression, cognitive impairment and physical frailty; hence, justifying the association between depression and cognitive frailty.

A possible explanation for the common co-occurrence of these three conditions is their common underlying pathophysiology marked by increased plasma levels of cortisol and inflammatory factors such as interleukin-6 [50]. However, the literature has been inconclusive and further research is required to explore the common molecular mechanisms of depression, cognitive impairment and physical frailty. Aside from the common pathophysiology, exacerbating effects of the common manifestations of these three conditions could also play a role in their co-occurrence. For instance, depression would lead to reduced social support and interaction, reduced physical activity and diminished self-care all of which could contribute to developing both physical frailty and cognitive impairment. On the other hand, physical frailty and cognitive impairment can exacerbate depression through the same mechanism. This indicates a potential negative feedback loop [49, 50]. Further research regarding this topic could help shed light on the relationship between these three conditions.

Disability is another factor associated with cognitive frailty. In this study we demonstrated that individuals with disability in more IADL items were more likely (OR 1.68, CI 1.44–1.96) to have cognitive frailty. This is consistent with findings from several cross-sectional studies [37, 53, 54]. Shimada et al. [53] indicated that inability to perform instrumental activities of daily living (IADL) would significantly increase the risk of cognitive frailty (OR 2.63). In a separate study, Choi et al. [20] demonstrated that individuals with cognitive frailty were more likely to have disability, and social support and satisfaction would reduce the risk of disability in these individuals. The association between disability and cognitive frailty has also been demonstrated in longitudinal studies [16, 55–57]. In a meta-analysis study, Zhang et al. [10] determined that older adults with cognitive frailty were three times more likely (OR 3.16) to have disability compared to those without cognitive frailty. Therefore, disability can be considered as both a risk factor and an adverse consequence of cognitive frailty.

Understanding the underlying pathophysiology of cognitive frailty is critical for development of intervention strategies and prevention of adverse health outcomes. However, to this date, research regarding this topic hasn’t been conclusive. Due to the higher incidence of dementia in older adults with cognitive frailty, it was initially hypothesized that cognitive frailty could be incipient dementia. Kocagoncu et al. [58] examined this hypothesis by comparing brain MRI images of older adults with cognitive frailty against those with Alzheimer’s disease. They concluded that neurophysiologic and brain structural changes in individuals with cognitive frailty differ from those with Alzheimer’s disease, hence ruling out the hypothesis that cognitive frailty could manifest early stages of dementia [58]. In a separate study Yushiura et al. [59] investigated brain structural changes and clinical features of cognitive frailty by examining brain MRI images of older adults with cognitive frailty. They observed more white matter lesions, lacunar infarcts, small-vessel disease lesions, micro-bleeds and reduced medial temporal lobe volume in individuals with cognitive frailty [59]. Future research in this field can further our knowledge on the underlying molecular pathway and etiology of cognitive frailty.

This study isn’t without limitations. Due to the cross-sectional nature of this study, the causal relationship between clinical factors and cognitive frailty cannot be established. Nevertheless, determining factors that are significantly associated with cognitive frailty in cross-sectional studies creates an important foundation upon which longitudinal studies can build and further investigate and establish causality. In this study we didn’t assess social factors and their effect on cognitive impairment. Some studies have indicated that social factors such as social support and participation can reduce the risk of adverse health outcomes; hence, taking such factors into account in future research can improve accuracy of the results. Moreover, the study population was limited to northern Iran, hence our findings may not necessarily extrapolate to different ethnic and geographical locations. Further research in this field is required in Iran to corroborate our findings.

Prevalence of cognitive frailty in this population of Iranian older adults was consistent with the estimated global prevalence of this condition. We demonstrated that older age, female gender, illiteracy, more comorbidities, depression and greater IADL disability were significantly associated with increased odds of cognitive frailty. Due to the pervasive impact of cognitive frailty on adverse health outcomes, future research should focus on developing screening tools and prevention strategies.

## Data Availability

The data used during the current study are available from the corresponding author on reasonable request.
